
JOURNAL INFORMATION
==============================
NLM Title Abbreviation: Behav Neurol
Journal ID: Behav Neurol
NLM Journal ID: 8914585
Journal ID: BN

Behavioural Neurology

ISSN: 0953-4180
EISSN: 1875-8584
Publisher: Wiley

ARTICLE INFORMATION
==============================
PMCID: PMC5434397
PMID: 21422567
DOI: 10.3233/BEN-2010-0307
Article ID: 956365
Article version: 1
Subjects: Research Article

The Automatic Pilot of the Hand is Unbalanced by Visual Neglect

McIntosh Robert D. 1 *
Rossit Stephanie 2
Malhotra Paresh 3
Butler Stephen H. 4
Harvey Monika 5
1 Human Cognitive Neuroscience University of Edinburgh Edinburgh UK
2 Department of Psychology University of Western Ontario Londen Canada
3 Division of Neurosciences and Mental Health Imperial College London London UK
4 School of Psychological Sciences and Health University of Strathclyde Glasgow UK
5 School of Psychology University of Glasgow Glasgow UK
*Robert D. McIntosh: r.d.mcintosh@ed.ac.uk
Print publication date: 2010
Electronic publication date: 2011 Mar 7
Volume: 23
Issue: 4
First page: 249
Last page: 251
Received 2011 Jan 31; Accepted 2011 Jan 31
Copyright: Copyright © 2010 Hindawi Publishing Corporation and the authors.
Copyright year: 2010
License: This is an open access article distributed under the Creative Commons Attribution License, which permits unrestricted use, distribution, and reproduction in any medium, provided the original work is properly cited.
License URL: https://creativecommons.org/licenses/by/3.0/

==============================
Behavioural Neurology 23 (2010) 249–251
DOI 10.3233/BEN-2010-0307
IOS Press

### The automatic pilot of the hand is unbalanced by visual neglect

Robert D. McIntosh[a,*], Stephanie Rossit[b], Paresh Malhotra[c], Stephen H. Butler[d] and Monika Harvey[e]
[a]*Human Cognitive Neuroscience, University of Edinburgh, Edinburgh, UK*
[b]*Department of Psychology, University of Western Ontario, Londen, Canada*
[c]*Division of Neurosciences and Mental Health, Imperial College London, London, UK*
[d]*School of Psychological Sciences and Health, University of Strathclyde, Glasgow, UK*
[e]*School of Psychology, University of Glasgow, Glasgow, UK*

The automatic pilot of the hand refers to the capacity for fast in-flight correction of reaching. It is often studied using ‘double-step’ reaching tasks, in which the target is jumped to a new location once the reach is underway. Target-directed corrections occur even if people are not instructed to follow the target (GO task), but are asked instead to stop the reach if they see the target jump (STOP task), suggesting that such corrections are a default visuomotor behaviour [9]. Uninstructed corrections in the STOP task were absent in a patient with optic ataxia following bilateral parieto-occipital lesions [9], implying that they depend upon the visuomotor dorsal stream.

Visual neglect after right hemisphere damage is most strongly associated with temporo-parietal lesions, inferior to the dorsal stream. It has thus been proposed that, in many patients with neglect, direct visuomotor behaviour should be free from the perceptual biases that characterise the syndrome [8]. Consistent with this, neglect does not seem to entail specific biases in reaching or grasping [1–3,5]. Moreover, patients may plan their reaches to avoid obstacles on the neglected side [6]; and a patient with visual extinction was shown to avoid obstacles, and to use online visual feedback from the hand, even when he could not report the relevant stimuli [7,11]. On the other hand, one study was able to isolate a motor-related component to neglect. Specifically, Mattingley et al. [4] demonstrated a retarded initiation of leftward movements into left hemispace in patients with neglect following inferior parietal lobe lesions.

We studied online visuomotor reactions to target jumps during double-step reaching in seven right-brain-damaged patients with neglect (RBDN; mean age 68.1 years, SD 9.5), eight right-brain-damaged controls without neglect (RDBC; mean age 62.5 years, SD 10.3), and eight healthy controls (HC; mean age 72.9 years, SD 4.0). Participants reached with the right index finger 40 cm forwards for a central target dot of 7 mm diameter, which remained static (70% of trials) or jumped at movement onset by 4 cm to the left (15% of trials) or right (15% of trials). In separate blocks of 200 trials, the instruction was to follow the target (GO task) or to stop the movement (STOP task) if the target jumped, with block order counterbalanced across participants. Movements were recorded from the finger at 108 Hz, using an electro-magnetic tracking system (MiniBIRD). For each participant, a spatial bandwidth for reaches to central targets was set at 2.81 SD either side of the average 2D hand path on static trials. For each frame of each jump trial, the reach was classed as corrected if its coordinates lay beyond the bandwidth of the static trials, *in the direction of the target jump*, being otherwise classed as uncorrected.

Patients with neglect reacted to rightward jumps as rapidly as controls, but were abnormally slow to react to leftward jumps; this was true even for RBDN

*Corresponding author: Robert D. McIntosh, Psychology, University of Edinburgh 7 George Square, Edinburgh, EH8 9JZ, United Kingdom. Tel.: +44 131 650 3444; Fax: +44 131 650 3461; E-mail: r.d.mcintosh@ed.ac.uk.

ISSN 0953-4180/10/$27.50 © 2010 – IOS Press and the authors. All rights reserved

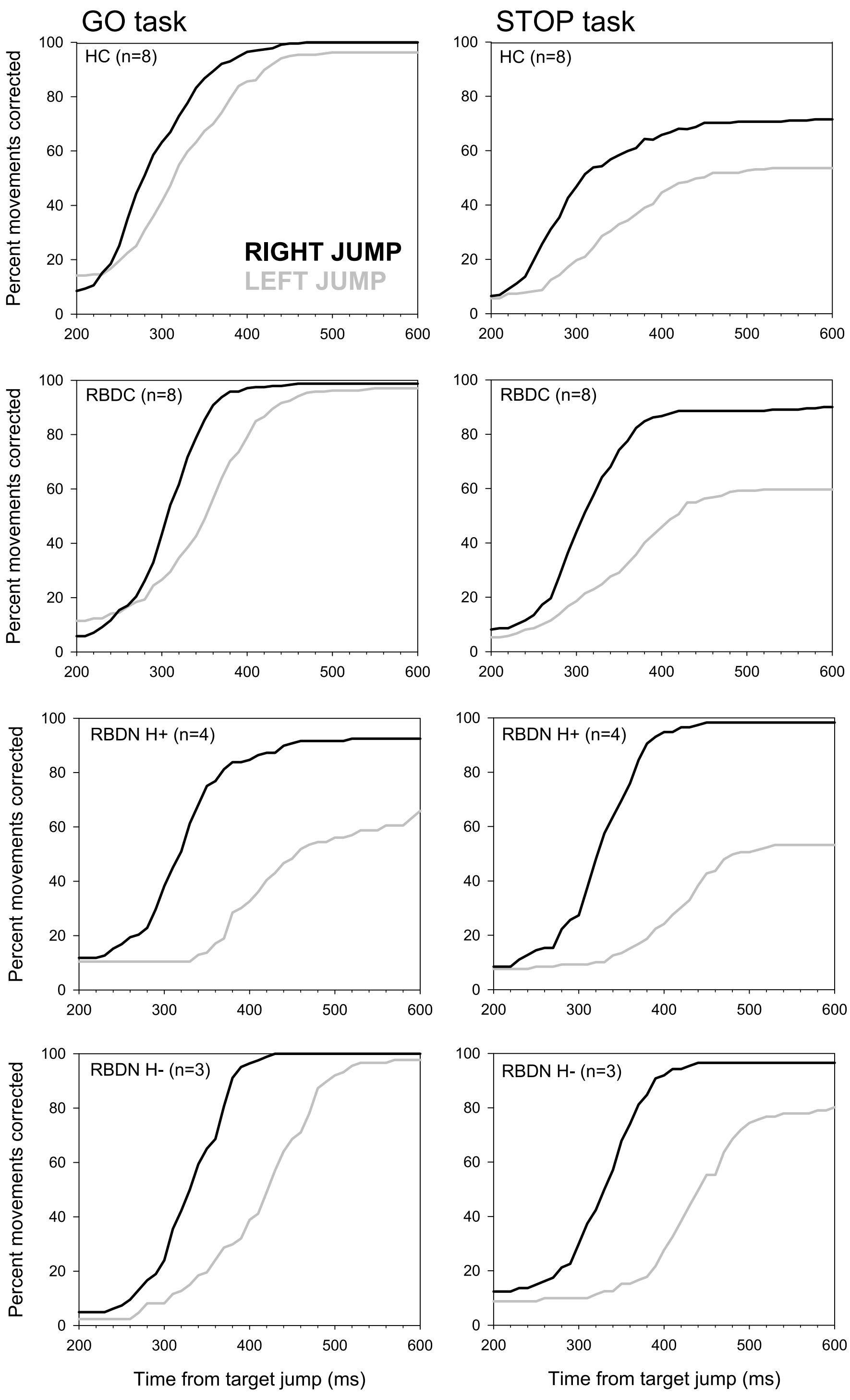

Fig. 1. Cumulative proportion of corrected responses on left jump (grey lines) and right jump (black lines) trials, by time from target jump, plotted up to 600 ms from target jump. Separate plots are shown for healthy controls (HC), right brain-damaged controls (RBDC), and right brain-damaged patients with neglect with hemianopia (RBDN H+) and without hemianopia (RBDN H-). The left column shows plots for the GO task, the right column for the STOP task. Both neglect sub-groups show abnormally late emergence of corrections to the left side. Nonetheless, corrections are made to the left even in the STOP task in which corrections are not instructed, implying integrity of the automatic pilot system, though with reduced responsiveness to the left side.

patients without left visual field deficits (assessed by computerised perimetry), as shown in Fig. 1. Moreover, an analysis of movement time for successfully corrected movements revealed a group by side interaction ($F_{2,19} = 6.63$, $p < 0.01$), reflecting differentially longer duration movements to the left than to the right in the RBDN (727 vs. 617 ms), as compared with the RBDC (639 vs. 581 ms) and HC (593 vs. 561 ms) groups. Nonetheless, neglect patients did make corrections to leftward target jumps, even in the STOP condition (Fig. 1). The occurrence of these uninstructed corrections suggest that the 'automatic pilot' system is functional in neglect; but it is unbalanced so that corrections to the left emerge abnormally slowly. This could conceivably be due to a retarded initiation of the corrective movement (a leftward movement into left hemispace [4]), but it seems more probable that the slowed correction to the left side is a consequence of inattention to the left, which reduces the salience of left-sided targets. We suggest that, although the automatic pilot system itself may be spared in neglect, its functioning is unbalanced by weakened attention to the left side. This implies shared attentional influences on vision-for-perception and vision-for-action, perhaps realised via top-down modulation of early visual areas.

An unexpected finding emerged from the analysis of the STOP responses themselves. The RBDN group had a dramatically impaired ability to stop their movements in response to a target jump to either side. A mixed-model ANOVA comparing successful stop rates across groups for left and right target jumps found a highly significant effect of group ($F_{2,19} = 7.27$, $p < 0.005$), but no effect of side ($p = 0.53$), and no interaction ($p = 0.54$). The neglect group successfully stopped their reaches on only 48% (SD 29) of jump trials, compared with 80% (SD 14) and 88% (SD 19) for RBDC and HC groups respectively. Although distinguishing the RBDN group overall, this impairment was unrelated to neglect severity within the group (correlation with BIT score: $r^2 = 0.08$). We propose that this finding reflects a non-lateralised deficit of response inhibition, which is not core to visual neglect, but which often accompanies the syndrome and may colour its expression [1].

### Acknowledgements

SR supported by Portuguese Foundation for Science and Technology grants (numbers SFRH/BD/23230/2005, SFRH/BPD/65951/2009).

### References

[1] S.H. Butler, S.R. Rossit, I.D. Gilchrist, C.J. Ludwig, B. Olk, R. Muir, I. Reeves and M. Harvey, Non-lateralised deficits in anti-saccade performance in patients with hemispatial neglect, *Neuropsychologia* **47** (2009), 2488–2495.

[2] M. Harvey, S.R. Jackson, R. Newport, T. Krämer, D. Llewlyn Morris and L. Dow, Is grasping impaired in hemispatial neglect? *Behavioural Neurology* **13** (2001), 17–28.

[3] M. Himmelbach and H.-O. Karnath, Goal-directed hand movements are not affected by the biased space representation in spatial neglect, *Journal of Cognitive Neuroscience* **15** (2003), 972–980.

[4] J.B. Mattingley, M. Husain, C. Rorden, C. Kennard and J. Driver, Motor role of human inferior parietal lobe revealed in unilateral neglect patients, *Nature* **392** (1998), 179–182.

[5] R.D. McIntosh, C.L. Pritchard, H.C. Dijkerman, A.D. Milner and R.C. Roberts, Prehension and perception of size in left visual neglect, *Behavioural Neurology* **13** (2001), 3–15.

[6] R.D. McIntosh, K.I. McClements, H.C. Dijkerman, D. Birchall and A.D. Milner, Preserved obstacle avoidance during reaching in patients with left visual neglect, *Neuropsychologia* **42** (2004a), 1107–1117.

[7] R.D. McIntosh, K.I. McClements, I. Schindler, T.P. Cassidy, D. Birchall and A.D. Milner, Avoidance of obstacles in the absence of visual awareness, *Proceedings of the Royal Society of London B* **271** (2004b), 15–20.

[8] A.D. Milner and R.D. McIntosh, Reaching between obstacles in spatial neglect and visual extinction, *Progress in Brain Research* **144** (2003), 213–226.

[9] L. Pisella, H. Grea, C. Tilikete, A. Vighetto, M. Desmurget, G. Rode et al., An 'automatic pilot' for the hand in human posterior parietal cortex: toward reinterpreting optic ataxia, *Nature Neuroscience* **3** (2000), 729–736.

[10] S. Rossit, P. Malhotra, K. Muir, I. Reeves, G. Duncan, K. Livingstone, H. Jackson, C. Hogg, P. Castle, G. Learmonth and M. Harvey, No neglect-specific deficits in reaching tasks, *Cerebral Cortex* **19** (2009), 2616–2624.

[11] T. Schenk, I. Schindler, R.D. McIntosh and A.D. Milner, The use of visual feedback is independent of awareness: evidence from visual extinction, *Experimental Brain Research* **167** (2005), 95–102.
